# Soldiers as subjects of medical research: Comments on Hassidim et al. on ethical standards of the Israel Defense Force

**DOI:** 10.1186/s13584-017-0136-5

**Published:** 2017-03-09

**Authors:** Asa Kasher

**Affiliations:** Department of Philosophy, Tel Aviv University, Tel Aviv, Israel

**Keywords:** Medical experimentation, Human subjects of experimentation, Nuremberg code, Helsinki principles, Israel defense force, Medical ethics, Conscripts as human subjects of experimentation, Israel defense force code of ethics

## Abstract

In 2008 a group of former soldiers of the Israel Defense Force (IDF) sued the Ministry of Defense and others, claiming they had suffered from medical problems that resulted from an IDF medical experiment in which they had participated in the 1970s. There was no compelling medical evidence with respect to causal relationships between their participation in the experiment and their later medical problems.

The President of the District Court, Justice Hila Gerstl, appointed me, with the consent of the parties, to write a deposition with respect to the ethical aspects of the case. My comments in the sequel rest on my deposition, applying not only to the case that had been under discussion but also to each and every case of experimentation. My arguments, strictly confined to the ethical aspects of the case, as opposed to the legal aspects and the debated facts, were not in favor of either party. As a result the state and the former soldiers reached an agreement approved by the court.

One of the major points made in that deposition is that the Nuremberg and Helsinki principles follow from those of medical ethics in general, except for the requirement to have an Institutional Review Board (IRB). A second major point is that under very strict conditions, more than what is usually required, soldiers may participate in medical experiments administered by their military force. However, new conscripts during their first months of their service should not take part in medical experimentation within their military force.

## Background

In their highly illuminating paper Hassidim et al. [1] describe major parts of the IDF MC (Israel Defense Force Medical Corps) practice of conducting experiments where the subjects are mostly soldiers during conscription service in the IDF. The paper is a survey and discussion of several ethical aspects of the experimentation practice. The purpose of the present comment is to add several ethical issues to the picture that emerges from the Hassidim et al. paper and briefly discuss them.

Our comments rest on our personal involvement in a certain discussion of ethical issues of experimentation within the framework of the IDF.

In 2008 a group of former soldiers of the IDF sued the Ministry of Defense and others, claiming they had suffered from medical problems that resulted from IDF medical experiment in which they had participated in the 1970s. There was no compelling medical evidence with respect to causal relationships between their participation in the experiment and their later medical problems. On the legal level of separate considerations of individual cases, plaintiffs would have lost, but when the State of Israel faces a phenomenon rather than a single case, an implicit principle of charity seems to be applied and the state is willing to shoulder responsibility without being blameworthy. Such a principle was used, for example, in the case of the IDF Navy divers who eventually suffered from cancer, allegedly but not statistically because of their training took place in an extremely polluted river (Shamgar Commission Report [2]). In the case here under discussion the state refused to reach an agreement on grounds of the financial burden it would have undertaken, had it agreed to compensate the suing former soldiers. It was argued that there are several thousands of soldiers who had participated in similar experimentation and would have been eligible to the same kind of compensation.

The President of the District Court, Justice Hila Gerstl, appointed me, with the consent of the parties, to write a deposition with respect to the ethical aspects of the case. My comments in the sequel rest on my deposition, applying not only to the case that had been under discussion but also to each and every case of experimentation. My arguments, strictly confined to the ethical aspects of the case, neither to the legal aspects nor to the debated facts, were not in favor of either party. As a result the state and the former soldiers reached an agreement approved by the court.

### Nuremberg and Helsinki Principles

The conditions set by the judges of the Nuremberg trials and the principles of the following Helsinki Declaration usually serve as starting points of discussions of experimentation that involves human subjects. Within the framework of legal discussions of such experimentation cases, the question naturally arises as to the extent to which these conditions and principles are legally binding. Israel does not have a law governing human experimentation but only formal regulations that went into effect at a certain point. What, then, is the force of the principles of Helsinki Declaration before the regulations brought them into effect in Israel?

In my deposition, I argued that the essence of the Nuremberg conditions and Helsinki principles rests on the values of medical ethics, from which no physician, whether during treatment or during investigation, is never exempted. By “values of medical ethics” I do not mean the famous four, viz. Autonomy, Beneficence, Non-Maleficence and Justice, because they are actually aspects of moral conduct that ought to be manifest under all circumstances, not only in medical practice. I take the basic values of medical ethics to be (a) Responsibility to protect human life and health, (b) Caring, in a certain practical sense, (c) Acting on scientific grounds, as well as (d) Professionalism and (e) Respect for human dignity. The deposition shows that all the ethical requirements of human experimentation but one stem from values (a)-(e), which means that they ought to be observed by every member of a medical team that administers experiments that involve human subjects.

The only exception is the requirement to have a procedure that approves proposals of human experimentation, without which not experiment may take place. This is an organizational requirement that significantly enhances ethical propriety of human experimentation. The paper by Hassidim et al. [1] is an example of how the IRB procedure can, and therefore should be improved for the sake of human subject protection, without thereby eliminating legitimate and most fruitful medical developments.

### The Spirit of IDF

The behavior of soldiers who serve in the IDF, both commanders and their subordinates, is under all circumstances governed by the ethics of the IDF. The values of the military ethics of the IDF, which appear in the “Spirit of IDF” document (at [3] http://www.idf/il), lead to consequences with respect to the required relationships between commanders and their subordinates. A principle relationship is that of Responsibility. A commander shoulders responsibility for the life and wellbeing of one’s subordinates, in combat, during exercises and under all other circumstances of military activity. Consequently, commanders are not expected to be utterly sequestered from what happens in the unit they command when human experimentation is planned or administered as the subjects are their subordinates. Commanders are never exempted from being responsible to and for the troops.

Since a genuine consent to participate in human experimentation requires freedom from any pressure, whether explicit or implicit, exerted by commanders to participate, a tension is thus created between the value of Responsibility of military ethics, which requires involvement of commanders, to a certain extent, and the value of Autonomy required for informed consent to participate in experimentation, which requires no involvement of commanders. I am not familiar with any attempt to obviate the tension by introducing a practice to be used by commanders when experimentation involving their troops are planned. I take it to be the combined duty of both the commanders and the medical corpse to develop such a practice, protecting both the sense of responsibility and the propriety of experimentation within the IDF.

### Basic training

In the case under discussion in the above-mentioned court procedure, some of the subjects were still at the entrance to the IDF. I take it to be an ethical mistake. At the entrance to the IDF and during basic training the independence of considerations required for genuine consent to participate in experimentation is not generally present. During the first periods of service a soldiers develops an understanding of the extent to which he remains a free citizen of the state, even though most aspects of his military life, such as profession, missions, practices and regular behavior, are governed by regulations, rules and commands. Before such a conception of partial independence is fully developed, soldiers should not participate in human experimentation.

The present regulations, as described by Hassidim et al. [1], allow participation of new conscripts in experimentation under some strict conditions. I think such an exception should be reconsidered. There are ways to gain medical knowledge pertaining to new conscripts without including them in experimentation, for example by administering the experiments to people of about the same age who are not new conscripts or by applying the results of experimentation in which soldiers participate after, say, six months of conscription. There are numerous such persons in Israel.

### Advanced training

An area that required special attention and perhaps new practices is that of experimentation the subjects of which are soldiers during exercises. Since military activity during exercises involves the commanders who conduct the exercises, sequestering them from the medical experimentation that is taking place by the medical teams during the exercises seems to be highly problematic. The tension is conspicuous between attempts to successfully achieve the goals of the exercises and attempts to successfully run the medical experiment.

Proper solutions of dilemmas should not take the form of opting for the more important horn of the dilemma and disregarding the other horn. A proper solution may involve setting a priority and supplement it with means for minimizing the damage caused to the value or end that is not of top priority under the circumstances. Consequently, planning experimentation the subjects of which are soldiers during exercises should be done in appropriate cooperation and ensuing coordination of commanders and medical teams.

### Support after participation in an experiment

An aspect of human experimentation that ought to be paid special attention when the subjects are soldiers is the support a soldier in active duty or a former soldier are going to get after the experimentation ended.

I have seen proposals for human experimentation, not just in the IDF, that included no clause of undertaking responsibility to the subjects of the experiment after it ends and they experience an effect that is, or at least they think it is a result of their participation in the experiment. This is morally and ethically wrong. It is wrong, first of all, because a moral person shoulders responsibility for the results of one’s actions and takes measures that guarantee that undesirable effects of one’s actions are properly attended. In the context of medical experimentation within the IDF it is wrong because the medical team as well as the soldier’s commanders in particular and the IDF in general are responsible for the life and wellbeing of the soldier, on grounds of medical ethics and the “The Spirit of IDF”. They should provide a soldier or a former soldier with practical help in treating problems one faces that are probably as a result of participation in experimentation.

The required practice won’t grant a soldier or a former soldier whatever one claims on ground of participation in experimentations, but two claims ought always to be fully respected. One is the thorough investigation of one’s medical condition in order to see whether one has been effected by the experimentation. Second is the best possible treatment of a problem in case there is reason to assume that it probably resulted from participation in experimentation.

Hassidim et al. [1] mention an arrangement with respect to classified research. An independent physician is identified to whom subjects have unlimited access before, during or after the medical study takes place. Concerns about potential side effects are mentioned as one of the possible issues to be discussed with such a physician. This is an excellent arrangement. I see no reason not to require a similar arrangement with respect to every experimentation within the IDF, at least for the period after the experimentation ended and a soldier or a former soldier needs access to a physician knowledgeable about the relevant experimentation. Such access might be needed many years after an experiment took place in which a person participated. Documentation is, therefore, required by regulation to be kept for a century.

## Conclusion

Our discussion shows that by and large the ethical principles that are meant to govern medical experimentation are actually consequences of the ethical principles of medical ethics in general.

Accordingly, soldiers may participate in medical experimentation under strict conditions related to informed consent as required by medical ethics and to responsibility of the IDF to the results of such experimentation as required by the Spirit of the IDF.

## Abbreviations

IDF MC: Israel defense forces medical corps
IDF: Israel defense forces
